# Perception of the acoustic environment during COVID-19 lockdown in Argentinaa)

**DOI:** 10.1121/10.0005131

**Published:** 2021-06-04

**Authors:** Ana L. Maggi, Jimena Muratore, Sara Gaetán, Mauricio F. Zalazar-Jaime, Diego Evin, Jorge Pérez Villalobo, María Hinalaf

**Affiliations:** 1 Center of Research and Transfer in Acoustics (CINTRA), Executing Unit of the National Scientific and Technical Research Council of Argentina (CONICET) and the National Technological University (UTN), Maestro M. Lopez esq. Cruz Roja Argentina, CP X5016ZAA, Córdoba, Argentina; 2 21st Century University, De los Latinos 8555, CP X5008, Córdoba, Argentina

## Abstract

The COVID-19 pandemic has significantly modified the behavior of societies. The application of isolation measures during the crisis resulted in changes in the acoustic environment. The aim of this work was to characterize the perception of the acoustic environment during the COVID-19 lockdown of people residing in Argentina in 2020. A descriptive cross-sectional correlational study was carried out. A virtual survey was conducted from April 14 to 26, 2020, and was answered mainly by social network users. During this period, Argentina was in a strict lockdown. The sample was finally composed of 1371 people between 18 and 79 years old. It was observed that most of the participants preferred the new acoustic environment. Mainly in the larger cities, before the isolation, mechanical sounds predominated, accompanied by the perception of irritation. Confinement brought a decrease in mechanical sounds and an increase in biological sounds, associated with feelings of tranquility and happiness. The time window opened by the lockdown offered an interesting scenario to assess the effect of anthropogenic noise pollution on the urban environment. This result offers a subjective approach, which contributes to understanding the link between individuals and communities with the environment.

## I. INTRODUCTION

The coronavirus (COVID-19) pandemic outbreak has generated a huge global impact since it began in late 2019. This situation consequently led government administrations to adopt containment measures such as lockdowns, unprecedented in recent history, which caused significant changes in human activities. The effects of these measures can be found on multiple levels, one of which is particularly relevant to this work: the notable decrease in general mobility and industrial activities.

The scientific research carried out during the COVID-19 outbreak initially focused on issues related to people's health and other topics such as the indirect impact of the pandemic on the environment were little studied at that time (Zambrano-Monserrate *et al.*, 2020). However, it soon became evident that the restrictions of many human activities meant a rich opportunity for the study of the impact of those activities on the environment and that the lockdown would make it possible to understand how the environment reacts to sharp reductions in anthropogenic activities. First, some authors reported changes in air and water quality, and it later was extended to other environmental factors from different regions of the world. Related to these factors, it was reported that the lockdown caused a reduction in the level of anthropogenic noise that altered the acoustic environment (Aletta *et al.*, 2020; Asensio *et al.*, 2020; Lecocq *et al.*, 2020; Derryberry *et al.*, 2020; Zambrano-Monserrate *et al.*, 2020).

Environmental noise is defined as an unwanted sound that could be generated by anthropogenic activities such as industrial or commercial activities, vehicular traffic, and loud music (Zambrano-Monserrate and Ruano, 2019). As early as 1973, Koczkur *et al.* (1973) identified traffic as one of the main sources of annoying noise. More recent studies such as Morillas *et al.* (2018) showed that this trend continues. Environmental noise is one of the main sources of discomfort for people and the environment, causing reduced quality of life, health problems, and alteration of the natural conditions of ecosystems. However, acoustic environments contain not only adverse sounds but also positive sounds. People tend to perceive natural sounds, like bird song or sounds from moving water, as positive components (Aumond *et al.*, 2017; Axelsson *et al.*, 2010). Acoustic environments in natural areas tend to be perceived as calm, pleasant, relaxing, and organized. In contrast, soundscapes heavily affected by traffic tend to be described as irritating, unpleasant, disturbing, and disorganized (Torija *et al.*, 2013).

The amount of work focused on the perception of the acoustic environment during the COVID-19 lockdown still is limited. The *Acoucité de France* carried out an investigation that included sound level measurements and an online questionnaire to obtain information on how people felt about the noise environment during the confinement. The responses show that, by comparing the situations before and during the lockdown, the perceived noise intensity decreased from 5.17 to 2.85 points on a scale of 0 to 10. In addition, their results showed that the sound environment had been profoundly modified. Noises from transportation and other human activities were reduced, while natural sounds became predominant during the period of confinement (Acoucité, 2020).

In Bulgaria, Dzhambov *et al.* (2021) conducted a study to understand how indoor soundscapes related to university students' self-rated health around the time that the country was under a state of emergency declaration caused by the COVID-19 pandemic. They found that greater exposure to mechanical sounds was consistently associated with worse self-rated health and that nature sounds correlated with higher restorative quality of the home.

In Italy, Bartalucci *et al.* (2021) carried out an online survey to obtain information on the context and characteristics of the house in which the participants lived, making a comparison of the lockdown and the pre-lockdown soundscapes. The results confirmed a general reduction of annoying sounds and an overall increase in the perception of nature sounds.

In Spain, Redel-Macías *et al.* (2021) investigated how the lockdown due to COVID-19 influenced people's perception of sound quality before and after lockdown through an online survey. Results showed that the global sound quality during lockdown improved drastically and that the perception of noise quality changed depending on the phase of the lockdown, the type of property, and the outside noise.

Aletta *et al.* (2020) suggest that future work should consider perceptual aspects of the urban acoustic environments experienced during the lockdown by analyzing cities of different sizes. Urban density could have significant effects on the distribution of some types of noise such as traffic noise (Wang and Kang, 2011 ).

In Argentina, on March 20, 2020, a general lockdown was established, which involved social, preventive, and obligatory isolation in order to avoid the circulation and spread of COVID-19. By issuing national decrees, it was established that people should refrain from going to their workplaces and could not travel along routes, roads, and public spaces. They could only make minimal and indispensable trips to stock up on cleaning supplies, medicines, and food. The only exception was for people affected by activities and services declared essential in the emergency: health, security, food industry, cleaning, and communication services, among others (Decree 297.2020; Argentina Ministry of Justice and Human Rights, 2020a). These measures have been dramatic and have had significant economic and social repercussions. They led to a reduction of more than 80% in mobility related to retail, recreation, and transport stations, as well as presence in parks (Google, 2020). Over time, isolation was managed through different phases through which the number of exempted activities and, as a consequence, the mobility of the population increased progressively. These levels of reduction in activity are unprecedented for our country and can be considered a valuable time window for contrasting acoustic environments under reduced anthropogenic noise pollution. The aim of this work was to characterize the perception of the acoustic environment during the COVID-19 lockdown of people residing in Argentina in 2020.

## II. METHOD

The research was carried out by the Sound Pollution and Hearing Conservation lines of the Center of Research and Transfer in Acoustics (CINTRA), Executing Unit of the National Scientific and Technical Research Council of Argentina (CONICET), and the National Technological University (UTN), Córdoba, Argentina.

A descriptive cross-sectional correlational study was carried out. A virtual survey was conducted from April 14 to 26, 2020, and was spread through social networks. During this period, Argentina was in phase 2, called “administered isolation” (validity of Decree 355/2020; Argentina Ministry of Justice and Human Rights, 2020b). In this phase, population mobility was reduced by 75% (Argentina Ministry of Health, 2020).

### A. Participants

Initially, the survey was answered by 1759 individuals.

The inclusion criteria were: age equal to or greater than 18 years, residence in Argentina, and completion of a form expressing agreement to voluntarily participate in the study.

The exclusion criterion was impossibility of residing in their usual home during the lockdown (this ensured that participants were aware of the previous acoustic environment). Participants who did not provide the name of their city of residence were also excluded from the sample.

The sample was finally composed of 1371 people between 18 and 79 years old, with a mean age of 37.04 (standard deviation = 11.97). Given that those who responded to the survey did so based on contact through social networks, we can presume that the population represented mainly consists of regular users of social networks.

The sample was organized according to the size of the cities (measured in the number of inhabitants), and the categories were designated according to a ranking indicating hierarchy (Erbiti, 2007):

Category I (CAT I): Towns and small cities, up to 49 999 inhabitants.
Category II (CAT II): Intermediate size agglomerations between 50 000 to 9 99 999 inhabitants.
Category III (CAT III): Agglomerations of 10 00 000 or more inhabitants.

### B. Techniques and instruments

The virtual questionnaire was designed *ad hoc* and was distributed *via* social networks, providing an online link^1^: The first section of the report dealt with socio-demographic data. The second section referred to the perceptual aspect of the acoustic environment of the house where the respondent was living during social isolation, involving the assessment of the type and level of noise near the house before and during the lockdown. The third section referred to the emotions associated with the acoustic environment before and during the lockdown.

### C. Ethical considerations

The study protocol was conducted according to the principles of the Declaration of Helsinki (World Medical Association, 2013). The questionnaire initially presented an informed consent text. Participants were informed that the survey was anonymous, free, and voluntary. Not answering it would not cause any harm. Answering it would not generate remuneration or any other monetary benefit.

### D. Data analysis

The statistical analysis was performed using SPSS^®^ software for Windows, version 20.0 (SPSS Inc., Chicago, IL). Chi-square test was used to analyze the association between main causes of annoying noise and city size; noise level of the places near the house before the lockdown and the pleasantness generated by the new acoustic environment (during the lockdown); prevalence of sounds in the house before and during the lockdown and city size; emotions related to the acoustic environment before and during the lockdown and city size. A Kruskal Wallis test was employed to compare differences in the ratings of the usual noise level between city size categories. This nonparametric analysis of variance was applied because a non-normal distribution of data was observed.

Finally, a McNemar test was applied to compare both the predominant sounds in the house before and during the lockdown, and the emotions linked to the acoustic environment before and during the lockdown. The significance value considered was *p* < 0.05.

## III. RESULTS

As can be seen in Table I, the sample was clearly biased towards a majority of female respondents, the age group of 26–40 years old was the one that gathered the most participants, and the predominant level of education was university. Regarding city size, the majority of participants belonged to cities with a population of one million or more (CAT III).

**TABLE I.** Demographic factors of the sample studied.

Demographic factors	Frequency	%
**Gender**		
Male	295	21.5
Female	1075	78.4
Other	1	0.1
**Age (years)**		
18–25	240	17.5
26–40	677	49.4
41–55	326	23.8
>55	128	9.3
**Education (highest level**)		
Primary	17	1.3
Secondary	444	32.4
Tertiary	143	10.4
University Degree	540	39.4
Postgraduate	226	16.5
**City size**		
Cat I (<=49.999 inhabitants)	241	17.6
Cat II (50.000–999.999 inhabitants)	387	28.2
Cat III (>=1.000.000 inhabitants)	743	54.2

### A. Main causes of annoying noise before the lockdown

**FIG. 1.**
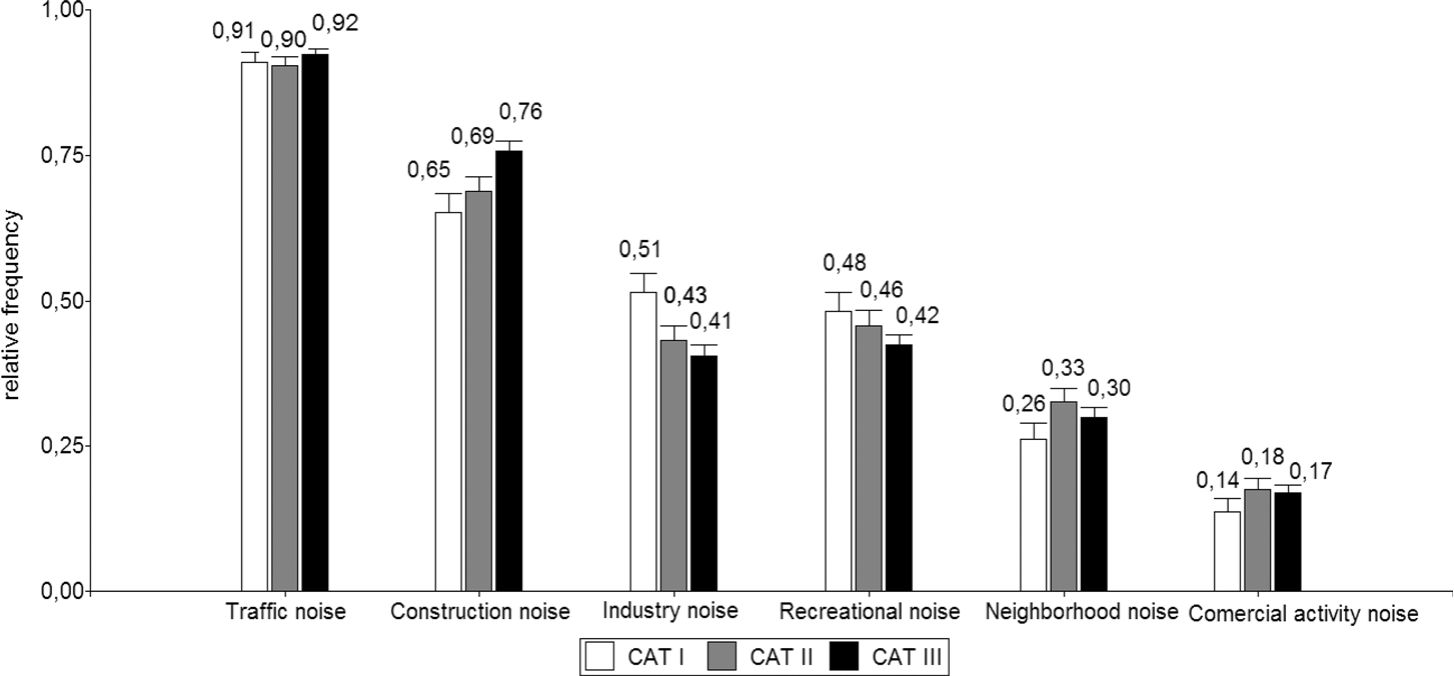
Main causes of annoying noise according to city size categories. CAT I, up to 49.999 inhabitants; CAT II, 50.000 to 999.999 inhabitants; CAT III, 1.000.000 or more inhabitants.

Figure 1 shows the main causes of annoying noise according to city size. Traffic was the most annoying, followed by construction, industry, and recreational noise. Applying Chi-square, a high correlation was found between city size and annoying noise from construction and industrial activities (*p* < 0.01). Construction activities were ranked as most annoying in cities of CAT III, while industrial activities were ranked as most annoying in CAT I.

**FIG. 2.**
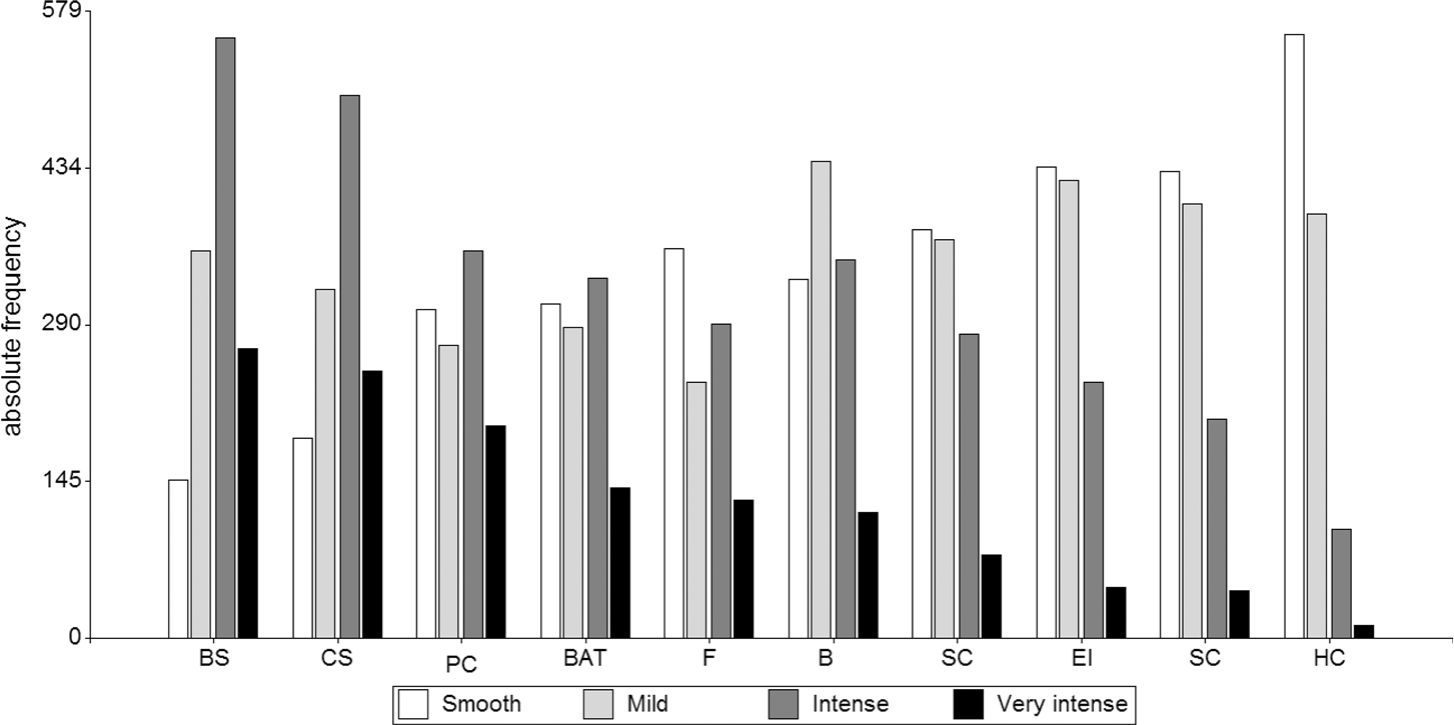
Perceived noise level from places near the house before the lockdown. BS, busy streets; CS, construction sites; PC, pubs and clubs; BAT, bus station, airports, train station; F, factories; B, bus stops; SC, shopping centers; EI, educational institutions; SC, sport centers; HC, health centers.

Figure 2 shows the usual noise level rating of the places near the house before the lockdown. The busy streets received the highest number of intense and very intense ratings, followed by construction sites, pubs, and clubs.

The Kruskall Wallis test was applied to compare differences when analyzing ratings of the usual noise level and the size of the cities. The most significant differences were found for busy streets (*p* < 0.01), followed by bus stops (*p* < 0.01), shopping centers (*p* < 0.01), and construction sites (*p* < 0.05). In particular, significant differences were observed between CAT I and CAT II and CAT I and CAT III. CAT III was reported as the loudest, exhibiting the highest mean value in most places, except for educational institutions and health centers that were perceived to have higher noise levels in CAT II and factories in CAT I.

### B. Annoying noise levels before lockdown and pleasantness of the acoustic environment during lockdown

When evaluating the pleasantness regarding the new acoustic environment generated by the lockdown, 74.5% of the participants stated that they felt the acoustic environment was more pleasant during the lockdown, while 18.7% did not care, and 6.8% liked it less.

A chi-square test was applied to identify the association between the noise level of the places near the house before the lockdown and the pleasantness generated by the new acoustic environment (during the lockdown). Significant associations were found for the noise level of the following places: bus stops (*p* < 0.01), busy streets (*p* < 0.01), shopping centers (*p* < 0.01), construction sites (*p* < 0.05), sport centers (*p* < 0.01), bus station, airports, train station (*p* < 0.05), health centers (*p* < 0.01), and educational institutions (*p* < 0.05). Descriptively, in most of the cases in which an association was observed, the group of participants who reported louder noise levels of the places near the house, before the lockdown, showed a higher percentage of pleasantness for the new acoustic environment than the group who reported lower noise levels. Health centers were the exception; the participants who reported very intense noise levels before the lockdown showed lower percentages of pleasantness for the new acoustic environment than the group who reported lower noise levels.

### C. Predominance of types of sounds before and during lockdown

**FIG. 3.**
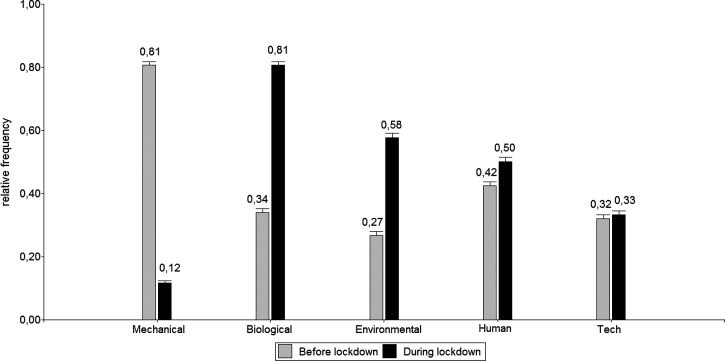
Predominance of types of sounds before and during lockdown.

Figure 3 compares the predominant sounds in the house before and during the lockdown. Before the lockdown, the predominant sounds were mechanical, while during the lockdown, they were biological. For this analysis, a McNemar test was applied. The types of sounds that showed significant differences before and during lockdown were: mechanical (*p* < 0.01), biological (*p* < 0.01), environmental (*p* < 0.01), and human (*p* < 0.01).

A chi-square test was applied to identify the association between the prevalence of sounds in the house before the lockdown and city size (Table II). Significant associations were found for mechanical, biological, and environmental sounds. Mechanical sounds were more predominant in CAT III. Biological and environmental sounds were more predominant in CAT I.

**TABLE II.** Percentage and *p*-value Chi-square analyzing the association between the predominance of types of sound and city size categories, before and during the lockdown. Statistically significant values (*p* < 0.05) are highlighted in gray.

Type of sounds	Before lockdown				During lockdown			
	CAT I	CAT II	CAT III	p-value	CAT I	CAT II	CAT III	*p*-value
Mechanical	68%	80%	85%	<0.01	10%	12%	12%	0.79
Biological	59%	36%	24%	<0.01	88%	83%	77%	<0.01
Environmental	41%	31%	20%	<0.01	65%	70%	54%	<0.01
Human	44%	44%	41%	0.45	49%	45%	53%	0.03
Tech	34%	32%	31%	0.67	37%	32%	33%	0.45

During the lockdown, significant associations were found for biological and environmental sounds with city size. Comparing before and during the lockdown, all categories showed a higher percentage of predominance of biological and environmental sounds during the lockdown. Biological sounds showed the greatest increase in CAT III and environmental sounds in CAT II. On the other hand, mechanical sounds, which showed significant association before the lockdown, did not show association with city size categories during the lockdown. In contrast, human sounds, which did not show association before the lockdown, showed significant association with city size during the lockdown, being more predominant in CAT III.

### D. Emotions associated with the acoustic environment before and during the lockdown

**FIG. 4.**
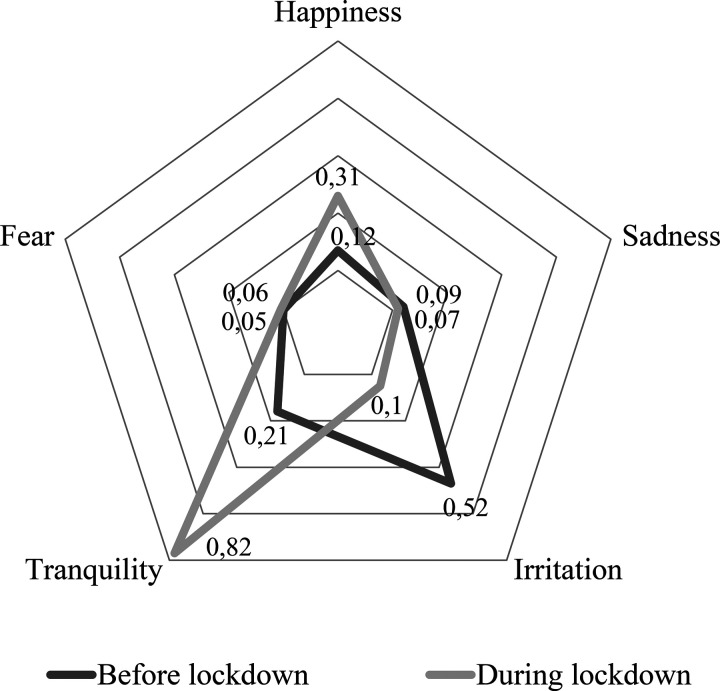
Emotions associated with the acoustic environment before and during the lockdown.

Figure 4 shows the emotions associated with the acoustic environment before and during the lockdown as reported by the participants. Before the lockdown, the most predominant emotion was irritation, while during the lockdown, a clear prevalence of tranquility is observed, followed by happiness. The McNemar test was used to determine significant differences between the emotions reported before and during lockdown. Those differences were significant for irritation (*p* < 0.01), tranquility (*p* < 0.01), and happiness (*p* < 0.01).

The chi-square test was applied to identify the association between emotions related to the acoustic environment and city size (Table III). Before the lockdown, significant associations were found for happiness (*p* < 0.01) and irritation (*p* < 0.01) with city categories. Happiness was predominant in CAT I. Irritation was predominant in CAT III. In contrast, during the lockdown, no emotion linked with the acoustic environment was significantly associated with the city size (*p* > 0.05).

**TABLE III.** Percentage and *p*-value Chi-square analyzing the association between emotions and city sizes categories, before and during the lockdown. Statistically significant values (*p* < 0.05) are highlighted in gray.

Emotions	Before lockdown				During lockdown			
	CAT I	CAT II	CAT III	p-value	CAT I	CAT II	CAT III	*p*-value
Happiness	20%	12%	10%	<0.01	37%	28%	30%	0.07
Sadness	9%	9%	8%	0.88	10%	6%	7%	0.20
Irritation	42%	49%	55%	<0.01	9%	11%	10%	0.74
Tranquility	24%	22%	19%	0.26	80%	80%	83%	0.46
Fear	3%	4%	7%	0.12	6%	6%	6%	0.99

## IV. DISCUSSION

The aim of this work was to characterize the perception of the acoustic environment during the COVID-19 lockdown of people residing in Argentina in 2020.

It was noted that the main cause of disturbing noise before the lockdown was traffic noise. Koczkur *et al.* (1973) identified traffic as one of the main sources of noise. More recent studies such as Morillas *et al.* (2018) showed that this trend continued before the pandemic.

When investigating the pleasantness regarding the acoustic environment during the lockdown, 74.5% of the participants stated that they liked the new acoustic environment more than the previous one, while 18.7% did not care, and 6.8% liked it less. In a survey conducted in France, it was reported that 63.4% of participants found the change in sound environment during lockdown pleasant due to COVID-19 (Acoucité, 2020). In a survey conducted in Spain, before the lockdown, the global sound quality was more defined as “Normal” (39%), while during the lockdown was more defined as “Really good” (51%) (Redel-Macías *et al.*, 2021).

In the present work, the types of sounds that showed significant differences before and during the lockdown were: mechanical, biological, environmental, and human. Before, the most predominant sounds were mechanical, and during, biological. This is in line with the results of Bartalucci *et al.* (2021), who confirmed a general reduction of annoying sounds and an overall increase in the perception of nature sounds. This modification in the composition of the sound environment and the changes in the hierarchy of sound sources was also evidenced in the work done by Acoucité (2020). Their results showed that biological sounds became predominant during lockdown. Transport noises and sounds related to other human activities were removed during the lockdown, giving rise to sounds that were there but remained barely perceptible.

In our research, we also found a significant association of the perceived noise level before lockdown with the pleasantness generated by the acoustic environment during lockdown. Those who previously perceived the noise level to be more intense had a tendency to feel the new acoustic environment was more pleasant. The positive contribution of natural sound sources was studied by Aumond *et al.* (2017), who mapped the pleasantness of the sound environment through perceptual evaluations and sound measurements on an urban walk in Paris. They found that the presence of birds was positively correlated with the feeling of pleasantness, and negatively correlated with the overall intensity level and the presence of traffic, concluding that birds were not present or were masked when traffic noise was present. Similar findings were found by Axelsson *et al.* (2010), observing that biological sounds are more pleasant.

With respect to the analysis we made on the emotions associated with the acoustic environment before and during the lockdown, before the lockdown, the predominant emotion was irritation, while during the lockdown, a clear predominance of tranquility was observed, followed by happiness. Similar findings were obtained in the research conducted by Acoucité (2020), in which the modification of the sound environment was accompanied by positive adjectives (calm, pleasant, peaceful). Cain *et al.* (2013) conducted a study in which they used semantic differential scale and principal component analysis to connect participants' feelings with sounds in urban spaces. They determined that the two main semantic descriptors were: “calm” and “vibration.” The characterization of parks with the presence of birds was located at the end of the axis that indicated higher levels of descriptors related to calm (tranquility, peace, relaxation, security), while streets with traffic noise were at the opposite end (agitated, stressed, scared, anxious). This linkage of sound environments with calm-related emotions can be compared with the findings of our research, in which before lockdown, mechanical sounds and irritation predominated, while during lockdown, biological sounds and tranquility predominated. This also coincides with Torija *et al.* (2013), who carried out a work whose objective was to develop an analysis of categorization and differentiation of soundscapes on the basis of acoustic and perceptual variables. They found that soundscapes in natural areas were perceived by the participants as calm, pleasant, relaxing, and organized. In contrast, soundscapes heavily affected by traffic were described as irritating, unpleasant, disturbing, and disorganized. In that sense, Dzhambov *et al.* (2021) conducted a study to understand how indoor soundscapes related to university students' self-rated health around the time that Bulgaria was under a state of emergency declaration due to the COVID-19 pandemic. They concluded that a restorative environment such as one supplying nature sounds and relatively free of mechanical sounds could contribute to personal coping resources needed to offset stress.

Cain *et al.* (2013) propose that the simple elimination of noise is not always appropriate and can generate anxiety. Franco *et al.* (2017) mention that similar consequences can generate the absence of natural sounds. It would have been useful to apply an instrument that evaluates anxiety in our research since the change in the acoustic environment was produced by a global pandemic.

In this study, we also analyzed the relationship between population size and variables linked to the perception of noise and emotions. Regarding the predominance of mechanical sounds before the lockdown, significant association with city size was observed, with a greater presence of this type of sounds in CAT III. Wang and Kang (2011) compared two typical cities (Manchester in the UK and Wuhan in China) and showed significant effects of urban density on the distribution of traffic noise. In our study, mechanical sounds, which showed significant association with city size before the lockdown, did not show association with city size during the lockdown.

Regarding the presence of biological and environmental sounds, before the lockdown, significant association between these types of sounds and city categories were observed, with greater presence in CAT I. Comparing before and during the lockdown, all categories showed a higher percentage of predominance of biological and environmental sounds during the lockdown. Biological sounds showed the greatest increase in CAT III and environmental sounds in CAT II. Derryberry *et al.* (2020) analyzed changes in bird songs during lockdown. They noted that the changes were smaller in rural areas than in urban areas, which would imply a greater decrease in noise levels in the latter.

As for the emotions associated with the acoustic environment according to the size of cities, before the lockdown, the perception of irritation was predominant for CAT III and the perception of happiness was predominant for CAT I. On the other hand, no emotion linked with the acoustic environment was significantly associated with the city size during the lockdown. These findings could be related to a greater perception of mechanical sounds in CAT III before the lockdown, since as mentioned, it is more likely that this type of sound, such as traffic, is associated with the perception of irritation (Cain *et al.*, 2013; Torija *et al.*, 2013). In the present research, mechanical sounds, which showed significant association with city size categories before the lockdown, did not show association during the lockdown. The perception of mechanical sounds became similar among city categories, which could explain the vanishing of differences in the perception of irritation and happiness between more and less populated cities.

With respect to this dimension, it is important to emphasize that we assume that the evaluation in terms of emotions evoked by acoustic characteristics is dynamic and is affected by multiple factors, such as personality, anxiety, uncertainties about the future, etc. (Kleinberg *et al.*, 2020). In that sense, we assume that the results found can be modified according to the moment they were made.

## V. CONCLUSIONS

The COVID-19 pandemic has significantly modified the behavior of societies. The application of isolation measures during the crisis resulted in changes in the acoustic environment. This work presents how the effect of anthropogenic noise reduction resulting from strict lockdown was perceived in Argentina. It was observed that most of the participants preferred the new acoustic environment. Mainly in the larger cities, before the isolation, mechanical sounds predominated, accompanied by the perception of irritation. Confinement brought a decrease in mechanical sounds and an increase in biological sounds, associated with feelings of tranquility and happiness.

These results were obtained in the initial instance of a pandemic that is ongoing and whose end is still unpredictable. While the results cannot be extended to society as a whole, we believe that they provide a valuable starting point for analyzing the changes experienced. The inclusion of the perceptual aspects linked to the acoustic environments during this period offers a subjective approach that cannot be captured by physical indicators. Taking a more holistic approach helps to understand the linkage of individuals and communities with the environment.

In relation to the limitations of this work, it can be mentioned that the type of sampling chosen biased the sample in terms of educational level (a large percentage of the participants had university education) and location (a large percentage of the participants were located in large cities), which led to a heterogeneous representation of the different points of the country. This could be due to the fact that respondents came mainly through social media announcements. However, it is valid to mention that the decision to invite people to participate through an online survey was based on the strict restrictions on activities resulting from the COVID19 lockdown implemented at the time this study was carried out. On the other hand, asking people to remember the acoustic environment could introduce a bias. However, we consider that the time elapsed from “before the lockdown” to the moment when the participants answered the questionnaire was short (25–36 days), reducing recall bias. For future research, it would be useful to ask participants about the number of people residing in the same place, the accessibility to quiet and green areas, the type and year of dwelling construction, and employment.

Finally, we consider that the time window opened by the lockdown offered an interesting scenario to assess the effect of anthropogenic noise pollution on the urban environment. Considering environmental acoustic quality, this analysis is valuable to estimate the potential improvements that could be obtained in urban conglomerates of different sizes. For instance, working on measures to control and mitigate noise sources such as traffic and construction. The pandemic, which has affected the world in 2020, arises as an opportunity for societies to review lifestyles and the impact they have on the planet.

## Footnotes

^1^ For more information, see https://www.investigacion.frc.utn.edu.ar/cintra/?pIs=3453.

## References

[1] Acoucité (2020). “ COVID-19 lockdown: Impact on the sound environment,” http://www.acoucite.org/confinement-covid-19-impact-sur-lenvironnement-sonore/ (Last viewed May 13, 2020) (in French).

[2] Aletta, F. , Oberman, T. , Mitchell, A. , Tong, H. , and Kang, J. (2020). “ Assessing the changing urban sound environment during the COVID-19 lockdown period using short-term acoustic measurements,” Noise Map. 7(1), 123–134. 10.1515/noise-2020-0011

[3] Argentina Ministry of Health (2020). “ Isolation administration phases,” https://www.argentina.gob.ar/coronavirus/aislamiento/fases (Last viewed October 26, 2020) (in Spanish).

[4] Argentina Ministry of Justice and Human Rights (2020a). “ Preventive and mandatory social isolation,” Decree 297/2020 DECNU-2020-297-APN-PTE, Official bulletin No. 15887/20 v. 20/03/2020, https://www.boletinoficial.gob.ar/detalleAviso/primera/227042/20200320 (accessed April 12, 2021) (in Spanish).

[5] Argentina Ministry of Justice and Human Rights (2020b). “ Preventive and mandatory social isolation,” Decree 355/2020 DECNU-2020-355-APN-PTE, Official Bulletin No. 16871/20 v, 11/04/2020, https://www.boletinoficial.gob.ar/detalleAviso/primera/227694/20200411 (Last viewed April 12, 2021) (in Spanish).

[6] Asensio, C. , Pavón, I. , and de Arcas, G. (2020). “ Changes in noise levels in the city of Madrid during COVID-19 lockdown in 2020,” J. Acoust. Soc. Am. 148(3), 1748–1755. 10.1121/10.0002008 33003833 PMC7857494

[7] Aumond, P. , Can, A. , De Coensel, B. , Botteldooren, D. , Ribeiro, C. , and Lavandier, C. (2017). “ Modeling soundscape pleasantness using perceptual assessments and acoustic measurements along paths in urban context,” Acta Acust united Ac. 103(3), 430–443. 10.3813/AAA.919073

[8] Axelsson, Ö. , Nilsson, M. E. , and Berglund, B. (2010). “ A principal components model of soundscape perception,” J. Acoust. Soc. Am. 128(5), 2836–2846. 10.1121/1.3493436 21110579

[9] Bartalucci, C. , Bellomini, R. , Luzzi, S. , Pulella, P. , and Torelli, G. (2021). “ A survey on the soundscape perception before and during the COVID-19 pandemic in Italy,” Noise Map. 8(1), 65–88. 10.1515/noise-2021-0005

[10] Cain, R. , Jennings, P. , and Poxon, J. (2013). “ The development and application of the emotional dimensions of a soundscape,” Appl. Acoust. 74(2), 232–239. 10.1016/j.apacoust.2011.11.006

[11] Derryberry, E. P. , Phillips, J. N. , Derryberry, G. E. , Blum, M. J. , and Luther, D. (2020). “ Singing in a silent spring: Birds respond to a half-century soundscape reversion during the COVID-19 shutdown,” Science 370(6516), 575–579. 10.1126/science.abd5777 32972991

[12] Dzhambov, A. M. , Lercher, P. , Stoyanov, D. , Petrova, N. , Novakov, S. , and Dimitrova, D. D. (2021). “ University students' self-rated health in relation to perceived acoustic environment during the COVID-19 home quarantine,” Int. J. Environ. Res. Public Health 18(5), 2538. 10.3390/ijerph18052538 33806377 PMC7967325

[13] Erbiti, C. (2007). “ Transformations of the Argentine urban system at the end of the 20th century: Challenges for land management,” in *Proceedings of the IV Seminar on Territorial Planning: Territorial Planning and Urban Problems*, November 6, 2007, Mendoza, Argentina, pp. 1–11 (in Spanish).

[14] Franco, L. S. , Shanahan, D. F. , and Fuller, R. A. (2017). “ A review of the benefits of nature experiences: More than meets the eye,” Int. J. Environ. Res. Public Health 14(8), 864. 10.3390/ijerph14080864 PMC5580568 28763021

[15] Google (2020). “ COVID-19 community mobility reports.2020,” https://www.google.com/covid19/mobility/ (Last viewed April 4, 2020).

[16] Kleinberg, B. , van der Vegt, I. , and Mozes, M. (2020). “ Measuring emotions in the covid-19 real world worry dataset,” arXiv:2004.04225.

[17] Koczkur, E. , Broger, E. D. , Henderson, V. L. , and Lightstone, A. D. (1973). “ Noise monitoring and a sociological survey in the city of Toronto,” J. Air Pollut. Control Assoc. 23(2), 105–109. 10.1080/00022470.1973.10469748 4687911

[18] Lecocq, T. , Hicks, S. P. , Van Note, K. , van Wijk, K. , Koelemeijer, P. , De Plaen, R. , Massin, F. , Hillers, G. , Anthony, R. , Apoloner, M. , Arroyo-Solórzano, M. , Assink, J. , Büyükakpınar, P. , Cannata, A. , Cannavo, F. , Carrasco, S. , Caudron, C. , Chaves, E. , Cornwell, D. , Craig, D. , den Ouden, O. , Diaz, J. , Donner, S. , Evangelidis, C. , Evers, L. , Fauville, B. , Fernandez, G. , Giannopoulos, D. , Gibbons, S. , Girona, T. , Grecu, B. , Grunberg, M. , Hetényi, G. , Horleston, A. , Inza, A. , Irving, J. , Jamalreyhani, M. , Kafka, A. , Koymans, M. , Labedz, C. , Larose, E. , Lindsey, N. , McKinnon, M. , Megies, T. , Miller, M. , Minarik, W. , Moresi, L. , Márquez-Ramírez, V. , Möllhoff, M. , Nesbitt, I. , Niyogi, S. , Ojeda, J. , Oth, A. , Proud, S. , Pulli, J. , Retailleau, L. , Rintamäki, A. , Satriano, C. , Savage, M. , Shani-Kadmiel, S. , Sleeman, R. , Sokos, E. , Stammler, K. , Stott, A. E. , Subedi, S. , Sørensen, M. , Taira, T. , Tapia, M. , Turhan, F. , van der Pluijm, B. , Vanstone, M. , Vergne, J. , Vuorinen, T. , Warren, T. , Wassermann, J. , and Xiao, H. (2020). “ Global quieting of high-frequency seismic noise due to COVID-19 pandemic lockdown measures,” Science 369(6509), 1338–1343. 10.1126/science.abd2438 32703907

[19] Morillas, J. M. B. , Gozalo, G. R. , González, D. M. , Moraga, P. A. , and Vílchez-Gómez, R. (2018). “ Noise pollution and urban planning,” Curr. Pollut. Rep. 4(3), 208–219. 10.1007/s40726-018-0095-7

[20] Redel-Macías, M. D. , Aparicio-Martinez, P. , Pinzi, S. , Arezes, P. , and Cubero-Atienza, A. J. (2021). “ Monitoring sound and its perception during the lockdown and de-escalation of COVID-19 Pandemic: A Spanish study,” Int. J. Environ. Res. Public Health 18(7), 3392. 10.3390/ijerph18073392 33805936 PMC8036401

[21] Torija, A. J. , Ruiz, D. P. , and Ramos-Ridao, A. F. (2013). “ Application of a methodology for categorizing and differentiating urban soundscapes using acoustical descriptors and semantic-differential attributes,” J. Acoust. Soc. Am. 134(1), 791–802. 10.1121/1.4807804 23862885

[22] Wang, B. , and Kang, J. (2011). “ Effects of urban morphology on the traffic noise distribution through noise mapping: A comparative study between UK and China,” Appl. Acoust. 72(8), 556–568. 10.1016/j.apacoust.2011.01.011

[23] World Medical Association (2013). “ World Medical Association Declaration of Helsinki: Ethical principles for medical research involving human subjects,” JAMA 310(20), 2191–2194. 10.1001/jama.2013.281053 24141714

[24] Zambrano-Monserrate, M. A. , and Ruano, M. A. (2019). “ Does environmental noise affect housing rental prices in developing countries? Evidence from Ecuador,” Land Use Policy. 87, 104059. 10.1016/j.landusepol.2019.104059

[25] Zambrano-Monserrate, M. A. , Ruano, M. A. , and Sánchez-Alcalde, L. (2020). “ Indirect effects of COVID-19 on the environment,” Sci. Total Environ. 728, 138813. 10.1016/j.scitotenv.2020.138813 32334159 PMC7169883

